# Sleep disturbances as risk factors for suicidal thoughts and behaviours: a meta-analysis of longitudinal studies

**DOI:** 10.1038/s41598-020-70866-6

**Published:** 2020-08-17

**Authors:** Lauren M. Harris, Xieyining Huang, Kathryn P. Linthicum, Chloe P. Bryen, Jessica D. Ribeiro

**Affiliations:** Department of Psychology, Florida State University, 1107 W. Call St., Tallahassee, FL 32306-4301 USA

**Keywords:** Psychology, Risk factors

## Abstract

In recent years, there has been a growing interest in understanding the relationship between sleep and suicide. Although sleep disturbances are commonly cited as critical risk factors for suicidal thoughts and behaviours, it is unclear to what degree sleep disturbances confer risk for suicide. The aim of this meta-analysis was to clarify the extent to which sleep disturbances serve as risk factors (i.e., longitudinal correlates) for suicidal thoughts and behaviours. Our analyses included 156 total effects drawn from 42 studies published between 1982 and 2019. We used a random effects model to analyse the overall effects of sleep disturbances on suicidal ideation, attempts, and death. We additionally explored potential moderators of these associations. Our results indicated that sleep disturbances are statistically significant, yet weak, risk factors for suicidal thoughts and behaviours. The strongest associations were found for insomnia, which significantly predicted suicide ideation (OR 2.10 [95% CI 1.83–2.41]), and nightmares, which significantly predicted suicide attempt (OR 1.81 [95% CI 1.12–2.92]). Given the low base rate of suicidal behaviours, our findings raise questions about the practicality of relying on sleep disturbances as warning signs for imminent suicide risk. Future research is necessary to uncover the causal mechanisms underlying the relationship between sleep disturbances and suicide.

## Introduction

Sleep is fundamental to survival^1–3^. A single poor night of sleep can result in mood changes^4^, worsening executive function^5^, and memory impairment^6^. Chronic sleep disturbances have been linked to increased risk for depression^7^, bipolar disorder^8^, and anxiety^9^. A growing body of research has also uncovered a link between sleep disturbances and suicide.

The notion that sleep disturbances contribute to suicide risk is gaining momentum. Sleep disturbance is commonly considered a warning sign for suicide^10^, and associations between sleep disturbances and suicidal thoughts and behaviours are consistently detected^11–15^. These associations are notable for several reasons. First, sleep problems are highly prevalent^16–18^. Approximately one-third of adults experience insomnia symptoms, with 6–10% meeting criteria for insomnia disorder^19^. To accurately assess risk, it is critical to determine the magnitude of the association between sleep disturbances and suicide. Second, sleep disturbances, unlike many other suicide risk factors, are modifiable. Sleep is already an intervention target in many mainstream therapeutic approaches, and treatments for sleep disturbances have been firmly established^20,21^. If sleep disturbances are reliably shown to be risk factors for suicide, sleep interventions could be leveraged as suicidality interventions. Third, sleep is an intervention target with relatively low stigma^22^, especially compared to suicidality^23^. Addressing suicidality by targeting sleep may increase the likelihood that at-risk individuals will seek treatment.

Until recently, research on the relationship between sleep and suicide has been predominantly cross-sectional^24^. Because risk factors must *precede* outcomes of interest^25^, cross-sectional evidence is insufficient to conclude that disturbed sleep is a risk factor for suicide. While longitudinal studies are filling this critical gap in the literature, results are mixed. Some studies have found large effects of sleep disturbances on suicide risk^26–28^, but others have found smaller^29–31^ or nonsignificant effects^32–34^. These discrepancies raise questions about the extent to which sleep disturbances confer risk for suicidal thoughts and behaviours. Moreover, these studies cannot provide information about causal mechanisms. Although risk factors are typically assumed to play a causal role in the outcome of interest, other variables may be responsible for observed longitudinal associations. For sleep disturbances to be considered *causal* risk factors for suicide, studies must examine whether manipulating sleep leads to systematic differences in suicide-related outcomes.

It is also unclear whether certain sleep disturbances are stronger predictors of suicidal thoughts and behaviours. Many studies focus on insomnia ^32,35,36^; others examine nightmares^37^, daytime sleepiness^33^, total sleep time^38^, and nonspecific or undifferentiated categories like “sleep problems”^30^. Even among studies that examine the same category of sleep disturbance, effect sizes range widely. Perhaps as a result of these inconsistent findings, the existing clinical guidelines are relatively nonspecific. Clinicians must rely on indicators like “unable to sleep” or “sleeping all the time” as warning signs for suicidal behaviours^10^. Given the seriousness of managing suicide risk, it is critical that clinical guidelines are clear so clinicians can make informed decisions about how to maintain their patients’ safety.

Measurement of sleep disturbances also varies across studies. Some studies use self-report scales^29,39^; others use clinical interviews^40^, and recent studies have begun to evaluate objective sleep parameters using actigraphy^41^ and polysomnography^42^. While novel methodologies refine the measurement of sleep disturbances, the comparative utility of objective versus subjective measures is unclear. Some evidence suggests that objective and subjective sleep measures are highly correlated^43^, whereas other studies find that individuals who present with subjective sleep complaints may not demonstrate objective evidence of disturbed sleep^44^. Therefore, their associations with suicide risk may be discrepant. Studies examining the relationship between objective sleep measures and suicide risk remain rare, but recent evidence indicates that both subjective and objective measures significantly predict risk, with similar effect sizes^41,42^. Given the cost and inconvenience of continuous sleep monitoring^45^, scalable subjective self-report measures may be preferable for routine monitoring of sleep disturbances.

Follow-up intervals also vary, ranging from one day^46^ to ten years^29^ for suicidal ideation, one month^47^ to eight years^33^ for suicide attempt, and one week^48^ to up to 50 years^49^ for suicide death. As clinicians are often tasked with identifying risk in the very short term, the most useful risk factors would accurately indicate imminent risk. It is critical to examine whether the effect of sleep disturbances on suicide risk varies with respect to time interval.

In short, the existing literature raises questions about the extent to which sleep disturbances serve as risk factors for future suicidal thoughts and behaviours. Although some have endeavoured to provide quantitative summaries of this literature^50^, these efforts have focused predominately on synthesizing cross-sectional research. Accordingly, it remains unclear whether sleep disturbances confer risk for suicidal thoughts and behaviours or whether they simply represent a correlate of those experiences. Advancing knowledge toward this end is critical to improving suicide prediction and prevention efforts.

The objective of this study is to substantively advance our understanding of the link between sleep disturbance and suicidal thoughts and behaviours. Using meta-analytic methods, the present study advances our knowledge in five ways. First, we will summarize the longitudinal literature on the relationship between sleep disturbances and suicide. Second, we will meta-analyse categories of sleep disturbances to determine whether certain categories (e.g., insomnia, nightmares, sleep quality) are stronger risk factors for suicide-related outcomes. Third, we will examine whether effects vary across outcomes (i.e., suicide ideation, attempts, or death). Fourth, we will evaluate the influence of potential moderators including study publication date, follow-up length, sample severity, and sleep measure type. Given recent methodological advances in the measurement of acute sleep disturbances, we hypothesized that more recent studies, particularly those which objectively measure sleep disturbances, would provide the most robust prediction. Although relatively little research has examined short-term prediction of suicide^51^, recent evidence indicates that shorter follow-up lengths may improve predictive accuracy^52^; therefore, we expected to detect stronger effects over shorter follow-up periods. Fifth, we will evaluate whether sleep disturbances serve as clinically useful predictors of suicidal thoughts and behaviours by contextualizing our findings in terms of the absolute risk of suicide-related outcomes.

## Methods

### Literature search

Our literature search was conducted as part of a larger meta-analytic effort^53^. Using identical methods and search terms, we updated the comprehensive literature search conducted by Franklin and colleagues^53^ to include articles published through October 31, 2019. Databases used were PubMed, PsycINFO, and Google Scholar. Search terms included variants of the words “longitudinal” (i.e., “longitudinal,” “longitudinally,” “predict,” “predicts,” “prospective,” “prospectively,” “future,” “later,”) and “suicide” (i.e., “self-injury,” “self-injurious,” “self-injurer,” “suicide,” “suicidal,” “suicidality,” “self-harm,” “NSSI,” “DSH,” “self-cutting,” “self-burning,” “self-poisoning”). Because many studies include measures of sleep disturbances even when they are not central to the study (e.g., a study about the effects of mood disorders on suicide may include information about insomnia), we intentionally did not constrain our search based on sleep-specific key words. We reasoned that this more comprehensive approach accordingly increased the likelihood that all potentially relevant articles would be captured.

### Inclusion and exclusion criteria

All articles were required to include at least one longitudinal analysis in which a sleep-related variable (i.e., any measure designed to assess sleep or sleep-related symptoms) predicted suicide ideation, attempt, or death. We focused on these outcomes for two reasons. First, we were interested in effects on suicidal thoughts and behaviours, which are self-directed and involve a nonzero intent to die. This excludes behaviours unrelated to suicidality, such as nonsuicidal self-injury, or mixed terms capturing both suicidal and nonsuicidal behaviours, such as deliberate self-harm. Second, we were interested in understanding specific effects on discrete suicide-related outcomes. Therefore, studies that collapsed these variables into a single measure (e.g., subsuming suicidal ideation and attempts under a “suicidality” item) were excluded. All articles were required to be peer-reviewed published articles with an English language version available. We chose to include only published studies because we were interested in publicly available data that clinicians may use to make decisions.

Treatment studies were excluded, as treatment effects may influence risk factor effects. Systematic reviews and meta-analyses were also excluded. Studies which did not provide necessary statistical information were also excluded (i.e., insufficient data to calculate an odds ratio and its variance).

### Study selection

**Figure 1:**
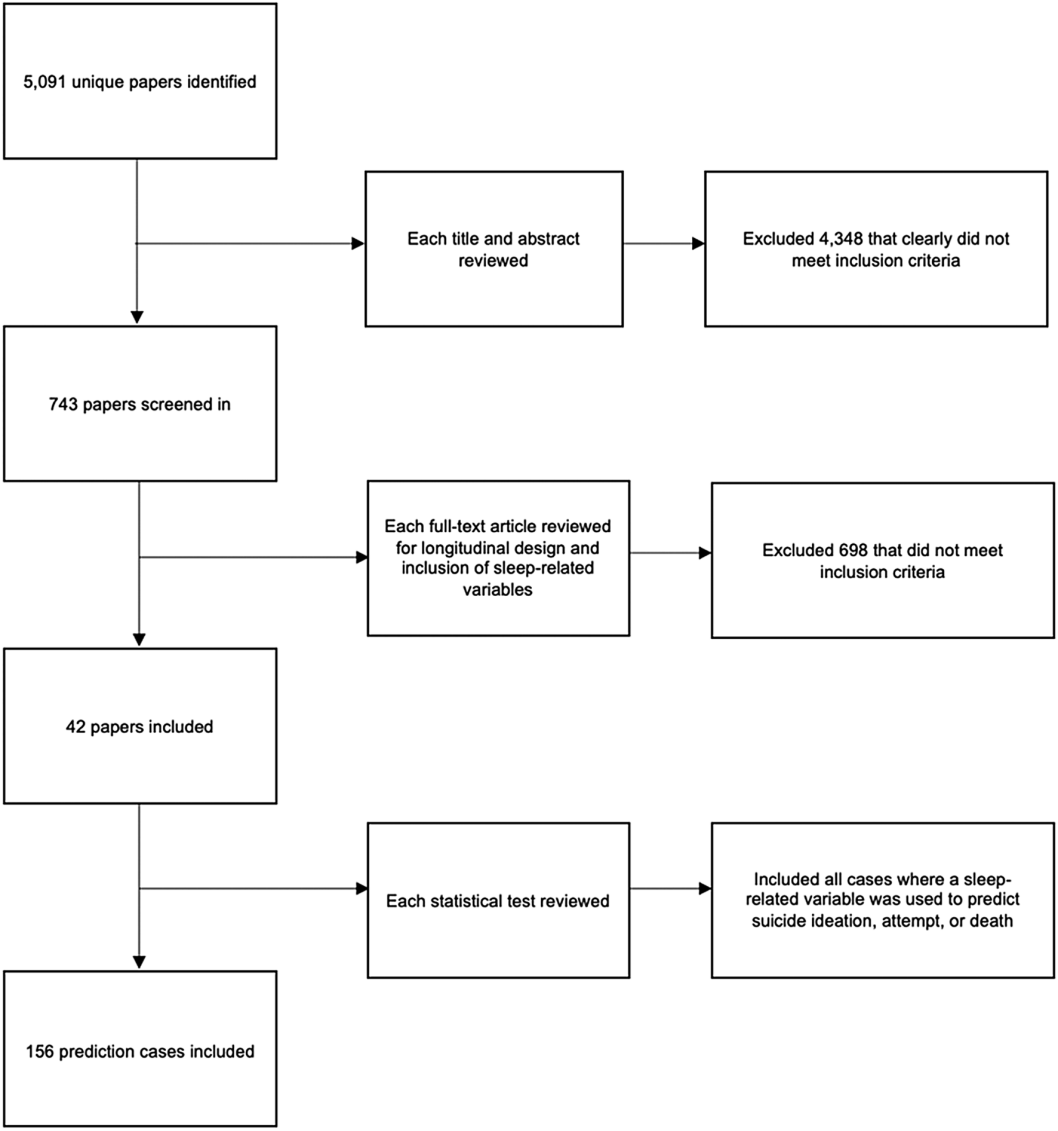
PRISMA flowchart^84^.

Our initial search yielded 5,091 unique articles published between 1965 and 2019. We screened in 743 papers for a full-text review. We retained 42 studies for our final analyses based on our inclusion criteria. Across studies, there were 156 unique effect sizes (see Fig. 1 for PRISMA flowchart; see Supplementary Materials for a full reference list of included studies).

### Data extraction and coding

The following data were extracted from each study: author, publication year, follow-up length in months, sample size, sample type (i.e., general population, clinical population, or participants recruited for a history of self-injurious or suicidal behaviours), sample age (i.e., “child/adolescent” if the study included only participants under 18 years of age at the start of the study, “mixed” if the study included both participants under and over 18 at the start of the study, or “adult” if all participants were over 18 years old), predictor (i.e., type of sleep disturbance), outcome (i.e., ideation, attempt, or death), sleep measure type (i.e., self-report, interview, actigraphy, or polysomnography), and relevant statistics. Any statistical test in which a sleep-related variable predicted suicide ideation, attempt, or death was retained as a “prediction case.”

Predictor variables varied across studies. To improve interpretability, we coded specific predictor variables into secondary broad predictor categories; see Supplementary Materials for a complete list. Initial codes for each article were determined by the first author. Each code was subsequently examined by two additional authors (CPB and KPL). All discrepancies were discussed until consensus and resolved. Outcomes were coded as suicide ideation, attempt, or death. One study^32^ included interrupted and aborted suicide attempts; these were coded as suicide attempts. Our pattern of findings remained unchanged when this study was excluded from analyses.

Meta-analyses often code for study quality, especially when the included studies contain a high degree of methodological variability. Compared to other meta-analyses, however, the present set of studies was relatively uniform, as they all shared a common core design (i.e., longitudinal prediction of a discrete suicide-relevant outcome). Because there are no objective criteria to assess study quality in this particular literature, we conducted moderator analyses of methodological differences (e.g., length of follow-up, predictor type, sample type, etc.) to examine the impact of how certain methodological differences may influence risk factor magnitude. This approach was consistent with the methods used in prior meta-analyses of suicide risk factor research^53–55^.

### Statistical analyses

All analyses were conducted using R^56^. Meta-analytic procedures were conducted using the metafor package^57^. We used odds ratios, which represent the odds of an event in one group compared to another, as our effect sizes. When odds ratios were not available, they were calculated from given data and summary statistics (e.g., 2 × 2 contingency tables, independent group means, risk ratios). If insufficient data were reported to compute an odds ratio and its variance (e.g., beta weights with no additional information, hazard ratios), that prediction case was excluded.

We used random effects models for all meta-analyses. Random effects models do not assume that a single, true effect exists, but that there will be a distribution of effects across studies. Due to the variability in predictors, outcomes, populations, and methodology, significant between-study heterogeneity was expected. Random effects models account for heterogeneity by relying on unconditional variance, which takes into account both sample size and variance between studies and weights effect sizes accordingly. Between-study heterogeneity was quantified using *I*^2^ tests. To improve the reliability of obtained estimates, only models including at least three effect sizes were run.

Publication bias was examined in several ways. We visually inspected funnel plots, which tend to be asymmetrical when publication bias is present and symmetrical when it is absent. Because visual inspection can be subjective, we also calculated Egger’s regression test as an objective index of funnel plot symmetry and used Duval and Tweedie’s trim and fill method to determine how many studies would be needed to make the funnel plot symmetrical. Classic and Orwin’s failsafe N analyses were conducted to estimate the robustness of observed effects.

Moderator analyses were conducted through a series of metaregressions using a random-effects model with unrestricted maximum likelihood estimation. Moderators included publication date, follow-up length, sample severity, and sleep measure type.

## Results

### Descriptive summary

**Figure 2:**
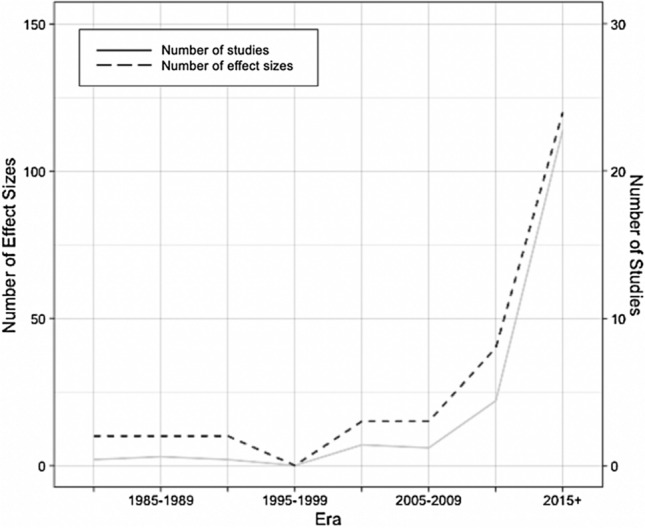
Number of studies and effect sizes over time.

Publication dates ranged from 1982 to 2019. The number of longitudinal studies examining the relationship between sleep disturbances and suicide has increased over time; most studies (N = 22; 52.38%) were published between 2015 and 2019 (Fig. 2). Suicide ideation was the most common outcome (k = 85; 54.49%), followed by attempt (k = 39; 25%) and death (k = 32; 20.51%).

Nearly half of prediction cases (k = 75; 48.07%) were drawn from community samples; 19.23% (k = 30) were drawn from clinical samples, and 32.69% (k = 51) were drawn from samples recruited for a history of self-injurious thoughts and behaviours. Most samples comprised only adults (k = 106; 67.95%); 29.49% of samples included only participants under 18 at the start of the study, and 2.56% (k = 4) included both participants under and over 18 at the start of the study.

Follow-up lengths ranged from 1 day to 50 years, with an average of 61.31 months (*SD* = 100.37, *Mdn* = 12). The follow-up lengths for suicide death cases (*M* = 197.40 months, range = 0.25–600, *SD* = 148.04, *Mdn* = 132) were longer on average than those for ideation (*M* = 23.61 months, range = 0.03–120, *SD* = 34.47, *Mdn* = 12) or attempt (*M* = 31.60 months, range = 0.03–100, *SD* = 30.44, *Mdn* = 12).

Insomnia was the most common predictor (k = 55; 35.26%), followed by unspecified “sleep problems” (k = 27; 17.31%), sleep duration (k = 16; 10.26%), and nightmares (k = 13; 8.33%). The majority of cases (k = 110; 70.51%) measured sleep disturbances via self-report, with the remainder using interviews (k = 29; 18.59%), actigraphy (k = 10; 6.41%), or polysomnography (k = 7; 4.49%).

### Overall prediction and publication bias

Overall prediction estimates reflect the pooled effects of all predictors on the outcome of interest. Odds ratio analyses included 156 prediction cases. The overall weighted odds ratio for all outcomes was 1.59 (95% CI 1.46–1.73). Between-study heterogeneity was high (*I*^2^ = 83.40%). See Table 1 for all random-effects results. Significant evidence of publication bias was not detected (Table 2).

**Table 1:** Random-effects results for each broad predictor category.

	Suicide ideation		Suicide attempt		Suicide death	
	n	OR [95% CI]	n	OR [95% CI]	n	OR [95% CI]
Insomnia	31	2.10 [1.83–2.41]	16	1.78 [1.38–2.29]	8	1.54 [1.04–2.29]
Nightmares	4	1.08 [0.61–1.91]	5	1.81 [1.12–2.92]	4	1.31 [0.83–2.06]
Sleep disturbances	6	1.61 [0.60–2.69]	3	1.85 [0.98–3.48]	–	–
Sleep duration	12	0.95 [0.70–1.29]	4	1.10 [0.75–1.68]	–	–
Sleep efficiency	4	1.26 [0.56–2.81]	–	–	–	–
Sleep problems	9	1.80 [1.30–2.50]	–	–	16	1.27 [0.97–1.66]
Sleep quality	6	1.74 [1.13–2.67]	3	1.35 [0.84–2.15]	–	–
Sleep onset latency	6	1.34 [0.75–2.42]	–	–	–	–
Tiredness	5	1.81 [1.00–3.27]	–	–	–	–

**Table 2:** Publication bias.

	Fail-safe N		Egger’s test of the intercept, z-value (*p*)	Duval and Tweedie’s trim and fill	
	Classic	Orwin’s		Missing cases	Adjusted OR
All outcomes	32,255	149	− 1.31 (0.19)	24	1.79
Suicidal ideation	13,011	81	− 2.00 (0.05)	17	1.92
Suicide plan	96	6	− 0.05 (0.96)	0	1.63
Suicide attempt	2021	36	0.83 (0.41)	1	1.54
Suicide death	378	32	0.02 (0.99)	6	1.18

#### Suicidal ideation

**Figure 3:**
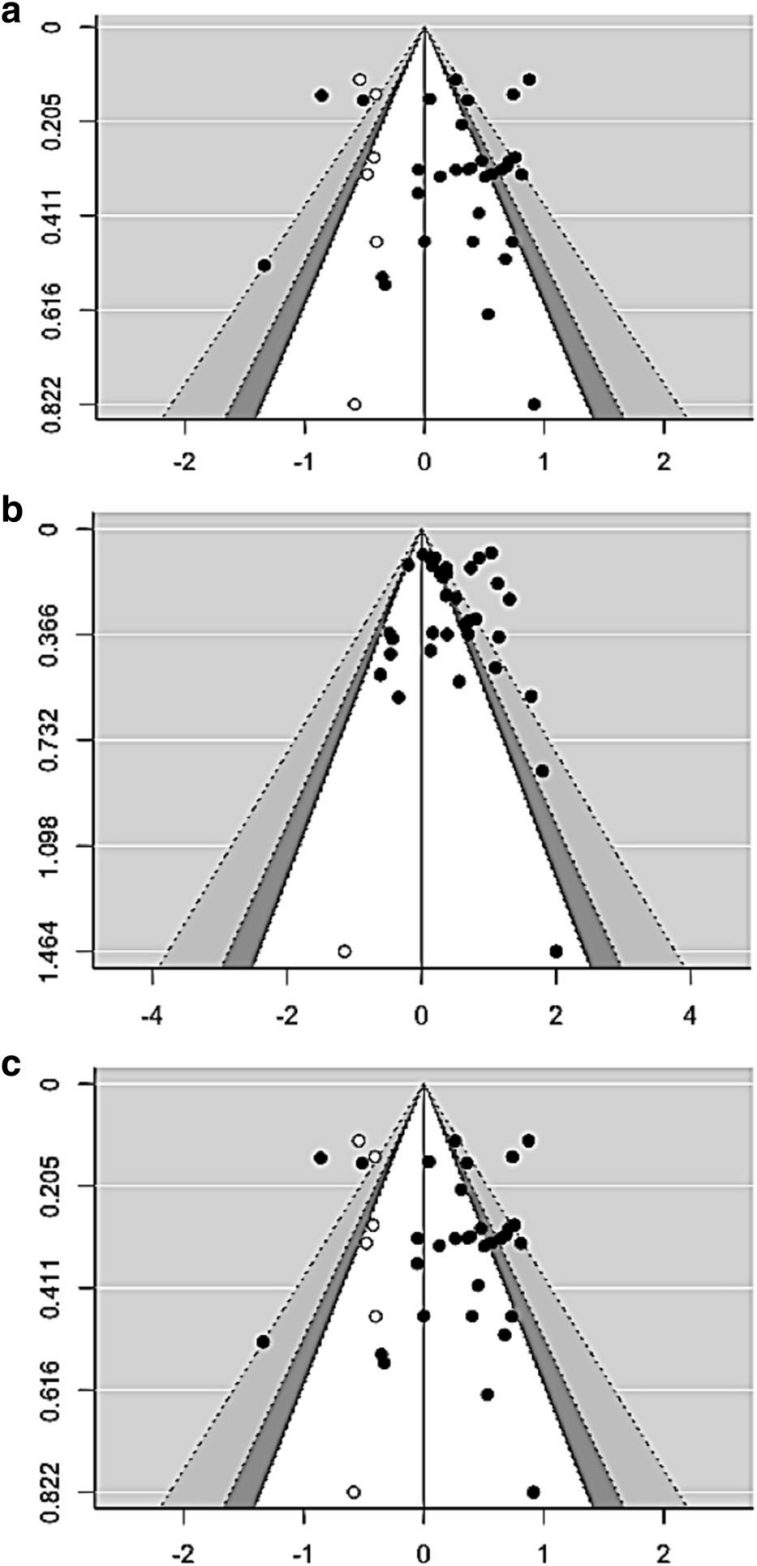
Funnel plots. (**a**) Suicide ideation, (**b**) suicide attempt, and (**c**) suicide death. Filled circles represent observed effects, and open circles represent imputed effects.

Odds ratio analyses for suicidal ideation included 84 prediction cases. Heterogeneity was high between studies (*I*^2^ = 83.91%). The overall weighted odds ratio was 1.73 (95% CI 1.54–1.94). While failsafe N analyses indicated that this was a robust effect, evidence of publication bias was detected via visual inspection of the funnel plot and Egger’s test of the intercept (Fig. 3a; Table 2).

#### Suicide attempt

Odds ratio analyses for suicide attempt included 36 prediction cases. The overall weighted odds ratio was 1.54 (95% CI 1.32–1.81). Between-study heterogeneity was high (*I*^2^ = 83.16%). While visual inspection of the funnel plot indicated possible publication bias, significant evidence for publication bias was not detected via Egger’s test of the intercept, and failsafe N analyses indicated this was a robust effect (Fig. 3b; Table 2).

#### Suicide death

Odds ratio analyses for suicide death included 32 prediction cases. The overall weighted odds ratio was 1.33 (95% CI 1.11–1.60). Between-study heterogeneity was high (*I*^2^ = 73.94%). Although visual inspection of the funnel plot indicated possible publication bias, no significant evidence for publication bias was detected via Egger’s test of the intercept, and failsafe N analyses indicated this was a robust effect (Fig. 3c; Table 2).

### Prediction by risk factor category

#### Insomnia

Insomnia significantly predicted suicidal ideation (OR 2.10 [95% CI 1.83–2.41], k = 31). Point estimates were smaller predicting suicide attempt (OR 1.78 [95% CI 1.38–2.29], k = 16) and suicide death (OR 1.54 [95% CI 1.04–2.29], k = 8).

#### Nightmares

Nightmares significantly predicted suicide attempt (OR 1.81 [95% CI 1.12–2.92], k = 5), but not suicide ideation (OR 1.08 [95% CI 0.61–1.91], k = 4) or death (OR 1.31 [95% CI 0.83–2.06], k = 4).

#### Sleep disturbances

Sleep disturbances did not significantly predict suicide ideation (OR 1.61 [95% CI 0.60–2.69], k = 6) or attempt (OR 1.85 [95% CI 0.98–3.48], k = 3). An insufficient number of cases were available to examine effects on suicide death.

#### Sleep duration

Sleep duration did not significantly predict suicide ideation (OR 0.95 [95% CI 0.70–1.29], k = 12), or attempt (OR 1.12 [95% CI 0.75–1.68], k = 4). An insufficient number of cases were available to examine effects on suicide death.

#### Sleep efficiency

Sleep efficiency did not significantly predict suicidal ideation (OR 1.26 [95% CI 0.56–2.81], k = 4). An insufficient number of cases were available to examine effects on suicide attempt or death.

#### Sleep problems

Sleep problems significantly predicted suicidal ideation (OR 1.80 [95% CI 1.30–2.50], k = 9). Effects were not significant for suicide death (OR 1.27 [95% CI 0.97–1.66], k = 16). An insufficient number of cases were available to examine effects on suicide attempt.

#### Sleep quality

Sleep quality significantly predicted suicidal ideation (OR 1.74 [95% CI 1.13–2.67], k = 6). Effects were not significant for suicide attempt (OR 1.35 [95% CI 0.84–2.15], k = 3). An insufficient number of cases were available to examine effects on suicide death.

#### Sleep-onset latency

Sleep-onset latency did not significantly predict suicidal ideation (OR 1.34 [95% CI 0.75–2.42], k = 6). An insufficient number of cases were available to examine effects on suicide attempt or death.

#### Tiredness

Tiredness did not significantly predict suicidal ideation (OR 1.81 [95% CI 1.00–3.27], k = 5). An insufficient number of cases were available to examine effects on suicide attempt or death.

### Moderator analyses

We report metaregression results in terms of Q_M_, the model sum of squares, which is a test of whether any of the regression coefficients in the model are significantly different from zero.

#### Publication date

Metaregression results indicated no significant effects of publication date on suicide ideation (Q_M_ [df = 2] = 0.31, *p* = 0.85), attempt (Q_M_[df = 2] = 1.15, *p* = 0.56), or death (Q_M_[df = 5] = 1.23, *p* = 0.94), indicating that effect sizes have remained stable over time.

#### Follow-up length

There was a significant effect of follow-up length for suicide ideation (Q_M_[df = 5] = 160.62, *p* < 0.0001), attempt (Q_M_[df = 5] = 39.71, *p* < 0.0001), and death (Q_M_[df = 5] = 13.29, *p* = 0.02). Effects were strongest when the follow-up length was 6 months or less for all outcomes (ideation OR 2.30 [95% CI 2.00–2.65]; attempt OR 2.48 [95% CI 1.33–4.62]; death OR 2.19 [95% CI 1.08–4.43]).

#### Sample severity

Moderation analyses revealed a significant effect of sample severity on suicide ideation (Q_M_[df = 2] = 21.61, *p* < 0.001), but not attempt (Q_M_[df = 2] = 0.20, *p* = 0.90) or death (Q_M_[df = 2] = 2.65, *p* = 0.27). Predicting ideation, effects were slightly stronger in community samples (OR 1.52 [95% CI 1.30–1.79]) and samples recruited for a history of self-injurious thoughts and behaviours (OR 1.45 [95% CI 1.17–1.80]) compared to other clinical populations (OR 0.78 [95% CI 0.57–1.05]).

#### Sleep measure type

Metaregression results indicated no significant effects of sleep measure type (i.e., self-report, interview, actigraphy, or polysomnography) on suicide ideation (Q_M_[df = 3] = 4.55, *p* = 0.21), attempt (Q_M_[df = 2] = 3.62, *p* = 0.16), or death (Q_M_[df = 1] = 0.25, *p* = 0.62). We also conducted post-hoc moderator analysis using each *specific* measure (e.g., specific self-report measures, interviews, and actigraphy/polysomnography measures) as a moderator. Our analyses revealed a significant effect of specific measure for suicide ideation (Q_M_[df = 13 ] = 54.50 , *p* < 0.001), but not attempt (Q_M_[df = 10] = 16.73, *p* = 0.08), or death (Q_M_[df = 12] = 11.88, *p* = 0. 46). For suicide ideation, effects were strongest when sleep disturbances were measured with the Insomnia Severity Index (OR = 1.55 [95% CI 1.07–2.23]) and the Adolescent Health Questionnaire (OR 1.63 [95% CI 1.10–2.42]).

## Discussion

Our findings indicate that sleep disturbances are statistically significant predictors of suicide ideation, attempt, and death. However, these effects were weak, at least as examined within the methodological constraints of the literature. Our results are consistent with a growing body of evidence which demonstrates that most commonly cited risk factors only weakly predict suicide^53,58,59^. Odds ratios for each outcome ranged from 1.33 to 1.73, and effects were consistent regardless of study publication date and type of sleep measure used (i.e., self-report, clinical interview, actigraphy, or polysomnography).

The literature on the longitudinal relationship between sleep and suicide has grown exponentially in recent years. Because the most recent meta-analysis of this literature was published nearly a decade ago and focused primarily on cross-sectional studies, the present study represents a critical step toward advancing our knowledge of the extent to which disturbed sleep confers risk for future suicidal thoughts and behaviours. Indeed, over half of the studies we uncovered in our review of the literature were published within the last 5 years (Fig. 2). Results indicated, however, that this increase in research has not corresponded with improved predictive accuracy, though it may have contributed to improved reliability of detected effects. We found that most studies examined the effects of disturbed sleep on suicide ideation, rather than suicidal behaviours (i.e., attempts or death). Moreover, very few studies examined proximal risk; the average follow-up length was over 5 years, and follow-up intervals were much longer on average for suicidal behaviours compared to suicidal ideation.

Effects varied depending on follow-up length, with slightly stronger effects observed over shorter follow-up periods (≤ 6 months); however, these effects remained weak. Prior evidence is mixed regarding the effects of follow-up length on risk factor strength. In a meta-analysis of hundreds of risk factors, no consistent patterns of predictive ability over different follow-up intervals were detected^53^. Given the fluctuating nature of sleep disturbances^60,61^ and suicidality^62,63^, it is possible that short-term follow-up windows yielded slightly stronger effects by producing more reliable measurement. Although these results indicate that it is sensible to focus on short-term prediction, studies with brief follow-up periods remain rare. Because suicide is fortunately a low base-rate event, it is difficult to detect statistically meaningful effects over brief intervals. Recent studies have used novel techniques to overcome this challenge, such as leveraging large, severe samples recruited online^64,65^. Online studies yield faster recruitment than in-person data collection and produce comparable results to in-person studies^66,67^. These methods may provide a fruitful path for future research.

Slightly stronger effects were detected in less severe samples. These findings are likely a methodological artefact. When studies rely on homogenous samples, the range of severity is restricted, making it difficult to detect significant differences from the reference group; in contrast, samples with high levels of heterogeneity capture both extremes of severity, making it easier to detect significant effects. Studies in clinical and self-injurious samples are also likely to control for additional risk factors, which may further reduce effect sizes. Other meta-analyses have found similar moderating effects of sample severity^54,58^. Despite statistically significant moderation, effects detected in less severe samples remained weak.

In addition to overall prediction, we examined the effects of specific sleep disturbances. The strongest effects were found for insomnia, which significantly predicted suicide ideation (OR 2.10 [95% CI 1.83–2.41]), and nightmares, which significantly predicted suicide attempts (OR 1.81 [95% CI 1.12–2.92]). Only insomnia significantly predicted suicide death (OR 1.54 [95% CI 1.04–2.29]). This pattern of findings is consistent with other meta-analytic evidence that the strongest predictive effects are typically observed for suicide ideation, followed by attempt and death (e.g., anxiety symptoms^58^; depression and hopelessness^54^); however, the evidence reliably demonstrates that even the strongest predictors are weak in absolute terms^53^. The majority of effects (76%) were nonsignificant, and for several predictors, there were too few cases to provide reliable estimates. Although additional cases may have allowed us to detect more reliable effects, it is unlikely that stronger effects would be detected, as overall estimates all achieved statistical significance yet remained weak.

This meta-analysis cannot provide direct insight into the relationship between sleep disturbances and suicide risk; it can only reflect the value of this relationship as examined within the methodological constraints of the literature. It is therefore important to consider limitations when interpreting these findings. First, although self-report was the most common way to assess sleep disturbances, nearly every study relied on different measures. The exceptions were three studies^32,65,68^ which used the Insomnia Severity Index (ISI), and two studies^35,69^ which used the Women’s Health Initiative Insomnia Rating Scale (WHIIRS). Other validated self-report measures included the Pittsburgh Sleep Quality Index^28,42^, the Adolescent Health Questionnaire^33^, the Youth Self Report questionnaire^70^, the Beck Depression Inventory^47^, and the Uppsala Sleep Inventory^71^. Multiple studies relied on single-item measures rather than validated questionnaires^30,72,73^. Although our moderation analyses did not reveal a significant effect of sleep measure *type* (i.e., self-report, interview, actigraphy, or polysomnography) on effect size, our post-hoc moderator analyses revealed that *specific* measures were statistically significant moderators for suicide ideation, with the strongest effects observed for the Insomnia Severity Index and the Adolescent Health Questionnaire. However, these effects remained weak (ORs 1.55 and 1.63, respectively). These two measures accounted for the largest proportion of effect sizes in our analyses; therefore, the significant moderation effect may reflect improved reliability as a consequence of their frequency of use. No significant effects were detected for suicide attempt or death.

Second, several sleep disorders were not represented in the existing literature, including sleep apnoea, restless leg syndrome, and narcolepsy. In fact, prior diagnoses of sleep disorders were sometimes used as exclusion criteria for study participation (e.g.,^42^). Although sleep disturbances may indicate the presence of a sleep disorder, it remains unknown whether their associations with suicide risk are distinct. Due to the lack of studies examining the longitudinal relationship between diagnosed sleep disorders and suicidal thoughts and behaviours, determining the extent to which they may confer risk for suicide is beyond the scope of the present meta-analysis; however, given prior meta-analytic evidence that diagnoses of particular disorders are limited in their ability to accurately predict suicide^53^, we reason that our pattern of results would have been comparable even with the addition of studies examining these disorders. Nevertheless, this represents an important area for future research.

Third, assessment windows varied substantially. Whereas the BDI assesses sleep disturbances over the last week, the ISI assesses the last two weeks, and the WHIIRS assesses the last four weeks. Some studies assessed sleep disturbances over the last year^72,74^. Consistent with a priori hypotheses, our moderator analyses demonstrated that effects were strongest over shorter intervals. Although relying on retrospective self-reported symptoms is common, doing so over longer periods of time is likely to be less valid and reliable than short-term assessment, as these methods may be affected by recall biases, especially for symptoms that occurred less recently^75^. These measurement issues may have contributed to the detection of less robust effects; however, the strongest short-term effect we detected (i.e., pooled effect of all sleep disturbances on suicide attempt over a follow-up period of 0–6 months) was still weak (OR = 2.48).

Fourth, although objective sleep measures address several limitations of subjective measures^76^, only three studies ^41,42,46^ used these methods, accounting for approximately 10% of effect sizes. It is possible that our failure to detect a significant effect of sleep measure type was due to the small number of studies using objective measures that fit our inclusion criteria. The included studies may not represent the effects of objective measures in general; sleep disturbances may emerge as stronger risk factors as additional studies are published. Because the effects of univariate predictors of self-injurious and suicidal behaviours are weak^53,77^, however, we reason that our results would be similar even with the inclusion of additional studies.

Our findings must also be evaluated with regards to clinical utility. According to the Centers for Disease Control and Prevention, the rate of suicide death in the United States is approximately 14.5 per 100,000. The strongest predictor of suicide death in this study was insomnia, which approximately doubles the risk of suicide death (OR 2.10). This increases the odds to 0.0003, representing a marginal improvement in predictive ability. Although sleep disturbances play a statistically significant role in predicting suicide, these results raise questions about the usefulness of designating sleep disturbances as suicide “warning signs,” at least when considered in isolation.

It is possible that methodological constraints account for the weak prediction estimates found in this study. However, our moderator analyses indicate this is unlikely. Even statistically significant influences on effects due to variations in follow-up length, sleep measure type, and sample severity did not meaningfully improve prediction. We accordingly reason that the most significant methodological issue is the focus on univariate-level prediction.

Our first recommendation for future research is to advance beyond examining the effects of sleep disturbances in isolation, and instead consider their function in the context of complex associations with other biopsychosocial factors. Studies which focus on individual sleep disturbances as predictors of suicide implicitly assume that this relationship is simple. Simple theories are cognitively manageable, but they stand in contrast to evidence demonstrating that it may be necessary to consider many biopsychosocial factors, combined in complex ways, to accurately predict suicide^65,78,79^. Results of the present study are consistent with complexity. Whereas no individual sleep disturbance was found to be particularly relevant to suicide, several were weak predictors. Although this does not mean that sleep disturbances are inconsequential for suicide risk, it indicates that the relationships between sleep disturbances and suicide are likely to be small, individual, and highly variable. Sleep disturbances are not necessary or sufficient in isolation for suicidality to arise. Continuing to examine *which* sleep disturbances contribute to suicide risk is unlikely to improve suicide prediction and prevention. Instead, a more promising path may be identifying *how* sleep disturbances can be incorporated into complex conceptualizations of suicide risk.

The aim of suicide science is not only to predict risk, but to uncover the causal processes underlying suicide and disrupt them. Our second recommendation is for future research to clarify causal mechanisms underlying the relationship between sleep and suicide. Longitudinal studies can establish risk factors, but they are unable to test causal hypotheses^25^. Experimental designs, in contrast, isolate the direct influence of risk factors; only experiments can be used to draw causal conclusions. Experiments are rare within the field of suicide research, but technological advances make it possible to safely and validly test causal hypotheses about suicide^80^. Experiments are more common in sleep research. The effects of experimentally induced sleep deprivation have been examined in the context of working memory^81^, pain perception^82^, and inflammation^83^. To our knowledge, no experiments to date have examined the influence of sleep disturbances on suicide. Future studies which leverage sleep deprivation paradigms in tandem with experimental approaches to studying suicide would advance our knowledge of whether sleep disturbances represent *causal* risk factors for suicide. This is a critical step toward designing interventions that directly target the causes of suicidal behaviour.

In sum, sleep disturbances increase risk for future suicide ideation, attempt, and death; however, these effects are weak in magnitude. The existing literature is methodologically constrained, but even with methodological advances, sleep disturbances are unlikely to emerge as strong univariate predictors of suicide. Although our results cast doubt on the utility of relying on sleep disturbances in isolation as warning signs for suicide, we look forward to future research examining the complex contributions of sleep disturbances to suicide risk. Future experimental studies are also needed to uncover potential causal mechanisms underlying the relationship between sleep disturbances and suicide.

## Supplementary information

Supplementary information.

## Data Availability

All relevant data are available upon reasonable request to the corresponding author.
