# Supplementary material for: Sleep disturbances as risk factors for suicidal thoughts and behaviours: a meta-analysis of longitudinal studies
Source: Sci Rep. 2020 Aug 17;10:13888. doi: 10.1038/s41598-020-70866-6 (PMC7431543; doi:10.1038/s41598-020-70866-6)
Supplement: Supplementary file 1 — Supplementary information. [file 41598_2020_70866_MOESM1_ESM.docx]

Supplementary Materials for:

**Sleep Disturbances as Risk Factors for Suicidal Thoughts and Behaviours: A Meta-Analysis of Longitudinal Studies**

Lauren M. Harris, BA; Xieyining Huang, MS; Kathryn P. Linthicum, MS; Chloe P. Bryen, BA; Jessica D. Ribeiro, PhD

Florida State University

Supplementary Table S1

*Specific predictors and broad predictor categories*

| Broad predictor | Specific predictors |
| --- | --- |
| Insomnia | Decreased sleep  Difficulty initiating sleep, difficulty falling asleep, trouble falling asleep  Difficulty maintaining sleep  Early morning awakening, early final awakening, waking up too early  HAMD delayed, initial, and/or middle insomnia  Insomnia, global insomnia, persistent insomnia  Insomnia and fatigue |
| Nightmares | Frequent nightmares  Nightmares 1x past year, nightmares several times past year, nightmares several times per month  Nightmares 1x/month, 1x/week, 3+/week  Nightmares occasionally, occasional nightmares  Nightmares often |
| Nonrestorative sleep | Insufficient sleep  Nonrestorative sleep |
| Sleep disturbances | Night wakings  Sleep disturbances  Wake after sleep onset*  Waking in the night |
| Sleep duration | Hours of sleep  Total sleep time objective*  Total sleep time subjective |
| Sleep efficiency | Habitual sleep efficiency  Sleep efficiency objective*  Sleep efficiency subjective |
| Sleep problems | Bedtime resistance  Comorbid insomnia and nightmares  Heightened arousal  Losing sleep because of worry  Need for sleep (decrease)  Nervous tension: difficulty sleeping  Severe sleeping problems  Sleep problems sometimes, almost every night, often  Sleep variability  Sleep (increase) / sleeping more than usual  Trouble sleeping |
| Sleep quality | Objective sleep quality*  Subjective sleep quality  Polysomnography N3%*  Polysomnography REM%*  Sleep quality fair, poor, very poor |
| Sleep-onset latency | Sleep onset delay  Sleep latency  Sleep onset latency objective*  Sleep onset latency subjective |
| Tiredness | Daytime sleepiness  Daytime sleepiness 1st, 2nd, 3rd, and 4th quartiles  Feeling tired nearly all the time  Overtired  Tiredness |

*Note*. All measures are subjective unless otherwise specified. An asterisk (*) signifies use of an objective measure.

**Studies Included in Meta-Analysis**

1. Allan, N. P. *et al.* Insomnia and suicidal ideation and behaviors in former and current U.S. service members: Does depression mediate the relations? *Psychiatry Research* **252**, 296–302 (2017).
2. Ballard, E. D. *et al.* Nocturnal Wakefulness Is Associated With Next-Day Suicidal Ideation in Major Depressive Disorder and Bipolar Disorder. *J. Clin. Psychiatry* **77**, 825–831 (2016).
3. Berglund, M. Suicide in Alcoholism: A Prospective Study of 88 Suicides: I. The Multidimensional Diagnosis at First Admission. *Arch Gen Psychiatry* **41**, 888 (1984).
4. Berglund, M. & Nilsson, K. Mortality in severe depression: a prospective study including 103 suicides. *Acta Psychiatrica Scandinavica* **76**, 372–380 (1987).
5. Bernert, R. A., Hom, M. A., Iwata, N. G. & Joiner, T. E. Objectively Assessed Sleep Variability as an Acute Warning Sign of Suicidal Ideation in a Longitudinal Evaluation of Young Adults at High Suicide Risk. *The Journal of Clinical Psychiatry* **78**, e678–e687 (2017).
6. Bernert, R. A., Turvey, C. L., Conwell, Y. & Joiner, T. E. Association of Poor Subjective Sleep Quality With Risk for Death by Suicide During a 10-Year Period: A Longitudinal, Population-Based Study of Late Life. *JAMA Psychiatry* **71**, 1129 (2014).
7. Bjørngaard, J. H., Bjerkeset, O., Romundstad, P. & Gunnell, D. Sleeping Problems and Suicide in 75,000 Norwegian Adults: A 20 Year Follow-up of the HUNT I Study. *Sleep* **34**, 1155–1159 (2011).
8. Blumenthal, S., Bell, V., Neumann, N. U., Schuttler, R. & Vogel, R. Mortality and Rate of Suicide of First Admission Psychiatric Patients: A 5-Year Follow-Up of a Prospective Longitudinal Study. *Psychopathology* 50–56 (1989).
9. Borg, S. E. & Stahl, M. Prediction of suicide: a prospective study of suicides and controls among psychiatric patients. *Acta Psychiatrica Scandinavica* **65**, 221–232 (1982).
10. Britton, P. C., Ilgen, M. A., Rudd, M. D. & Conner, K. R. Warning signs for suicide within a week of healthcare contact in Veteran decedents. *Psychiatry Research* **200**, 395–399 (2012).
11. Eikelenboom, M., Beekman, A. T. F., Penninx, B. W. J. H. & Smit, J. H. A 6-year longitudinal study of predictors for suicide attempts in major depressive disorder. *Psychol. Med.* **49**, 911–921 (2019).
12. Fawcett, J. *et al.* Time-related predictors of suicide in major affective disorder. *American Journal of Psychiatry* **147**, 1189–1194 (1990).
13. Graves, P. & Thomas, C. B. Habits of nervous tension and suicide. *Suicide and Life-Threatening Behavior* **21**, 91–105 (1991).
14. Joo, J., Hwang, S. & Gallo, J. J. Death Ideation and Suicidal Ideation in a Community Sample Who Do Not Meet Criteria for Major Depression. *Crisis* **37**, 161–165 (2016).
15. Kim, H. H. The impact of online social networking on adolescent psychological well-being (WB): a population-level analysis of Korean school-aged children. *International Journal of Adolescence and Youth* **22**, 364–376 (2017).
16. Kivelä, L. *et al.* Longitudinal course of suicidal ideation and predictors of its persistence – A NESDA study. *Journal of Affective Disorders* **257**, 365–375 (2019).
17. Li, S. X. *et al.* Sleep Disturbances and Suicide Risk in an 8-Year Longitudinal Study of Schizophrenia-Spectrum Disorders. *Sleep* **39**, 1275–1282 (2016).
18. Littlewood, D. L. *et al.* Short sleep duration and poor sleep quality predict next-day suicidal ideation: an ecological momentary assessment study. *Psychol. Med.* **49**, 403–411 (2018).
19. Liu, X. *et al.* Nightmares Are Associated With Future Suicide Attempt and Non-Suicidal Self-Injury in Adolescents. *J. Clin. Psychiatry* **80**, (2019).
20. Liu, X. *et al.* Daytime sleepiness predicts future suicidal behavior: a longitudinal study of adolescents. *Sleep* **42**, (2019).
21. Lubin, G. *et al.* Suicide in the Israeli Military: Case-Controlled, Prospective and Retrospective Study. 8 (2018).
22. Mars, B. *et al.* Predictors of future suicide attempt among adolescents with suicidal thoughts or non-suicidal self-harm: a population-based birth cohort study. *The Lancet Psychiatry* **6**, 327–337 (2019).
23. Meir, P., Alfano, C. A., Lau, S., Hill, R. M. & Palmer, C. A. Sleep patterns and anxiety in children interact to predict later suicidal ideation. *Children’s Health Care* **48**, 372–393 (2019).
24. Pien, F.-C. *et al.* Changes in Quality of Life After a Suicide Attempt. *West J Nurs Res* **38**, 721–737 (2016).
25. Ribeiro, J. D., Huang, X., Fox, K. R., Walsh, C. G. & Linthicum, K. P. Predicting Imminent Suicidal Thoughts and Nonfatal Attempts: The Role of Complexity. *Clinical Psychological Science* **7**, 941–957 (2019).
26. Ribeiro, J. D. *et al.* Sleep problems outperform depression and hopelessness as cross-sectional and longitudinal predictors of suicidal ideation and behavior in young adults in the military. *Journal of Affective Disorders* **136**, 743–750 (2012).
27. Ribeiro, J. D., Yen, S., Joiner, T. & Siegler, I. C. Capability for suicide interacts with states of heightened arousal to predict death by suicide beyond the effects of depression and hopelessness. *Journal of Affective Disorders* **188**, 53–59 (2015).
28. Roane, B. M. & Taylor, D. J. Adolescent insomnia as a risk factor for early adult depression and substance abuse. *Sleep* **31**, 1351–1356 (2008).
29. Sandman, N. *et al.* Nightmares as predictors of suicide: an extension study including war veterans. *Sci Rep* **7**, 44756 (2017).
30. Schneider, B., Philipp, M. & Müller, M. J. Psychopathological predictors of suicide in patients with major depression during a 5-year follow-up. *European Psychiatry* **16**, 283–288 (2001).
31. Schneider, B. *et al.* Living alone, obesity, and smoking increase risk for suicide independently of depressive mood findings from the population-based MONICA/KORA Augsburg cohort study. *Journal of Affective Disorders* **152–154**, 416–421 (2014).
32. Sjöström, N., Hetta, J. & Waern, M. Persistent nightmares are associated with repeat suicide attempt. *Psychiatry Research* **170**, 208–211 (2009).
33. Stange, J. P. *et al.* Specific mood symptoms confer risk for subsequent suicidal ideation in bipolar disorder with and without suicide attempt history: multi-wave data from STEP-BD. *Depression and Anxiety* **33**, 464–472 (2016).
34. Start, A. R., Allard, Y., Adler, A. & Toblin, R. Predicting Suicide Ideation in the Military: The Independent Role of Aggression. *Suicide Life Threat Behav* **49**, 444–454 (2019).
35. Suh, S. *et al.* Longitudinal Course of Depression Scores with and without Insomnia in Non-Depressed Individuals: A 6-Year Follow-Up Longitudinal Study in a Korean Cohort. *Sleep* **36**, 369–376 (2013).
36. Tanskanen, A. *et al.* Nightmares as predictors of suicide. *Sleep* **24**, 845–848 (2001).
37. Tuvey, C. L. *et al.* Risk factors for late-life suicide: a prospective, community-based study. *American Journal of Geriatric Psychiatry* **10**, 398–406 (2002).
38. Wakefield, J. C. & Schmitz, M. F. Feelings of worthlessness during a single complicated major depressive episode predict postremission suicide attempt. *Acta Psychiatr Scand* **133**, 257–265 (2016).
39. Wang, H. E. *et al.* Pre-deployment insomnia is associated with post-deployment post-traumatic stress disorder and suicidal ideation in US Army soldiers. *Sleep* **42**, (2019).
40. Wong, M. M. & Brower, K. J. The prospective relationship between sleep problems and suicidal behavior in the National Longitudinal Study of Adolescent Health. *Journal of Psychiatric Research* **46**, 953–959 (2012).
41. Wong, M. M., Brower, K. J. & Zucker, R. A. Sleep problems, suicidal ideation, and self-harm behaviors in adolescence. *Journal of Psychiatric Research* **45**, 505–511 (2011).
42. Zuromski, K. L., Cero, I. & Witte, T. K. Insomnia symptoms drive changes in suicide ideation: A latent difference score model of community adults over a brief interval. *Journal of Abnormal Psychology* **126**, 739–749 (2017).
